# A national survey to assess breast cancer awareness among the female university students of Pakistan

**DOI:** 10.1371/journal.pone.0262030

**Published:** 2022-01-21

**Authors:** Iltaf Hussain, Abdul Majeed, Imran Masood, Waseem Ashraf, Imran Imran, Hamid Saeed, Anees Ur Rehman, Furqan K. Hashmi, Fahad Saleem, Muqarrab Akbar, Muhammad Omer Chaudhry, Jamshid Ullah, Muhammad Fawad Rasool

**Affiliations:** 1 Department of Pharmacy Practice, Faculty of Pharmacy, Bahauddin Zakariya University Multan, Pakistan; 2 Department of Pharmacy Practice, Islamia University Bahawalpur, Bahawalpur, Pakistan; 3 Department of Pharmacology, Faculty of Pharmacy, Bahauddin Zakariya University, Multan, Pakistan; 4 University College of Pharmacy, University of the Punjab, Allama Iqbal Campus, Lahore, Pakistan; 5 Department of Pharmacy Practice, University of Balochistan, Quetta, Pakistan; 6 Department of Political Science, Bahauddin Zakariya University, Multan, Pakistan; 7 School of Economics, Bahauddin Zakariya University, Multan, Pakistan; 8 Department of Medical Lab Technologies, University of Haripur, Haripur, Pakistan; Aga Khan University, PAKISTAN

## Abstract

The incidence of breast cancer is increasing in Pakistan as well as globally. Awareness of women about breast cancer plays a cornerstone role in its early detection, better management, and prevention. Keeping this in mind, a cross-sectional study was carried out to assess the awareness of female university students about breast cancer’s risk factors, signs and symptoms, and breast cancer examination. The data was collected from female university students studying in Pakistan. A total of 774 participants completed the survey and recorded their responses on an online pre-tested self-administered questionnaire. Only 29.8% of the participants have identified breast cancer history in their first-degree relatives as a risk factor. Moreover, 14.1% of the participant considered that the use of oral contraceptives for more than 5 years can increase the risk of developing breast cancer. In addition, inward pulled nipple, wounds around the nipple, and abrupt changes in the breast size were considered as the sign and symptoms of breast cancer by 25.2%, 25.7%, and 31.7% of the participants, respectively. Moreover, only 20.9% of the participants identified the correct year for starting breast cancer examination and 44.4% of the respondents marked that mammography should be initiated after 40 years. Overall, the university female students of Pakistan were poorly aware of breast cancer’s risk factors, signs and symptoms, and breast examination. This study has highlighted the need for initiation of aggressive strategies regarding breast cancer awareness in both the literate and illiterate female population of Pakistan.

## Introduction

Breast cancer is a disease in which breast cells start an uncontrolled growth and produce an undifferentiated cell mass [1]. The American Cancer Society (ACS) broadly divided breast cancer into in-situ and invasive breast cancer [2]. The in-situ breast cancer starts in the milk duct and stay it locally. This type of breast cancer includes ductal carcinoma in-situ (DCIS) which is considered as a non-invasive or pre-invasive breast cancer. While invasive breast cancer has the potential to spread into the surrounding breast tissues and includes invasive ductal carcinoma and invasive lobular carcinoma [1, 2].

It has been reported that increasing age is highly associated with the incidence of breast cancer [3]. Additionally, women having a family history of breast cancer, are more prone to the development of this cancer [4]. Reproductive factors like early menarche, late menopause, late age at first pregnancy, and low parity can also increase the breast cancer risk. The delay in menopause each year increases the risk of breast cancer by 3% [5–7]. Moreover, the exogenous and endogenous estrogen is significantly associated with the risk of breast cancer. The main source of exogenous estrogen is oral contraceptive and hormone replacement therapy. This medication results in enlarged and tender breasts [8]. The risk of breast cancer is slight higher in women using oral contraceptives as compared who never used them [5]. The use of this medication can over-stimulate breast tissue, and could increase the risk of breast cancer. Moreover, the higher fat and low fiber diet can play role in the development of breat cancer [3, 5, 9, 10].

Breast self-examination (BSE), clinical breast examination, and mammography are the most widely used screening techniques [11]. BSE is a screening method for early breast cancer detection that can be performed at home. This is a basic, cheap, straightforward, and efficient approach for examining breast tissue for physical or visual abnormalities. BSE improves women’s chances of treatment by enhancing their odds of survival [12]. Despite advancements in therapy, identifying breast cancer as early as possible is critical for improving health outcomes. Breast health education organizations recommend that all women begin practicing BSE regularly as soon as their breasts are completely formed. For example, the Maurer Foundation recommends that BSE be conducted at least once a month beginning at the age of 18 years. Women get familiar with their breasts as a result of such regular scrutiny and are thus more likely to identify any changes [13].

Globally, the ratio of breast cancer cases is increasing day by day and it is the second leading cause of mortality [11]. In 2020, the women diagnosed with breast cancer was 2.3 million and the associated deaths were 685,000. Breast cancer is the world’s most prevalent cancer, as there were 7.8 million women alive who were diagnosed with breast cancer at the end of 2020 [12]. As compared to other cancer, there are more disability-adjusted life years (DALYs) lost by women to breast cancer globally [13]. In Pakistan, the number of reported breast cancer cases was 25, 928 which accounted for 14.5% of all types of cancer in 2020 [14]. The risk of developing breast cancer has risen in Pakistan, with one in every nine women having a lifetime risk of being diagnosed with this cancer [15].

Awareness about the disease play important role in its early diagnosis. This awareness helps the population to avoid risk factors associated with the disease. A heightened awareness of breast cancer disease can aid the women in adherence to its screening guidelines. This adherence will lead the population to the early diagnosis of breast cancer that will help the healthcare provider for better management and also will increase the surveillance rate of the patients. The university female students were less touched in the previous reports from Pakistan, and they were limited to a single location and specific domain like some were focused on risk factors other were sign and symptoms and breast cancer examination [16–19]. Therefore, the current national survey of Pakistan was conducted to assess the understandings and awareness of female university students about breast cancer’s risk factors, signs and symptoms, and breast cancer examination.

## Material and method

### Study design and participants

A cross-sectional approach was used in the current study. The study participants were university female students that were enrolled in the different universities of Pakistan. The students that were Pakistani nationals, had ages greater than 18 years, and who gave their consent to participate were included in the study. The term undergraduate includes bachelor programs (BS), while the post-graduate includes master (MPhil/MS), and doctorate programs (Ph.D.) in the current study.

### Study instrument

A self-administered questionnaire was developed based on previous reports [20–22]. The questionnaire was composed of two parts. The first part comprised of demographic questions including age, education level, and residence area and province. While the second part comprised of questions regarding breast cancer risk factors, signs and symptoms, and the best time for breast examination. The domain which includes the risk factors were further divided into three sub-domains based on suggestions from the face validation and pilot study. The first sub-domain includes risk factors related to the history of breast cancer and benign diseases, exposure to radiation and use of medication (D1), the second sub-domain comprised of risk factors related to gynecology and obstetrics (D2), and the third sub-domain include the risk factors related to physical activities, and lifestyle (D3). The study instrument has been provided in the S1 File.

The questionnaire was validated through face validation followed by a pilot study (N = 75). The Cronbach’s alpha value of 0.81 was obtained, which indicated a good internal consistency.

### Data collection

A total of 774 respondents have completed the survey that enrolled in different universities located in four provinces (Khyber Pakhtunkhwa, Punjab, Sindh, and Balochistan) of Pakistan through an online survey. Google forms (Google LLC, California, United States) were used to create the pre-tested questionnaire, and a link was shared with participants via social media platforms (Facebook, WhatsApp, and E-mail). An online written informed consent was taken from each participant before recruitment in the study. Participant confidentiality was maintained throughout the study. The hard file was kept in the department lab locker and the soft file was maintained in the password-protected folder. The online data was collected as per the Checklist for Reporting Results of Internet E-Surveys (CHERRIES) guidelines [23, 24].

### Ethical considerations

The current study was reviewed and approved by the Department of Pharmacy Practice, Faculty of Pharmacy, BZU Multan (Reference No: Acad/PRAC/21/04) and conducted as per the Declaration of Helsinki. The current study was reported according to “The Strengthening the Reporting of Observational Studies in Epidemiology (STROBE)” guidelines [25].

### Statistical analysis

The statistical package for social science (SPSS) (IBM SPSS Statistics for Windows, Version 25.0. Armonk, NY: IBM Corp.) was used for data analysis. The descriptive statistics and cross-tabulation were performed and the frequency and percentage were calculated to illustrate categorical variables. The Chi-square test was applied to assess the association between study questions and education level. A p-value ≤ 0.05 was considered significant for all the statistical analysis. The Bonferroni correction was used to counteract with multiple comparisons.

## Results

**Fig 1:**
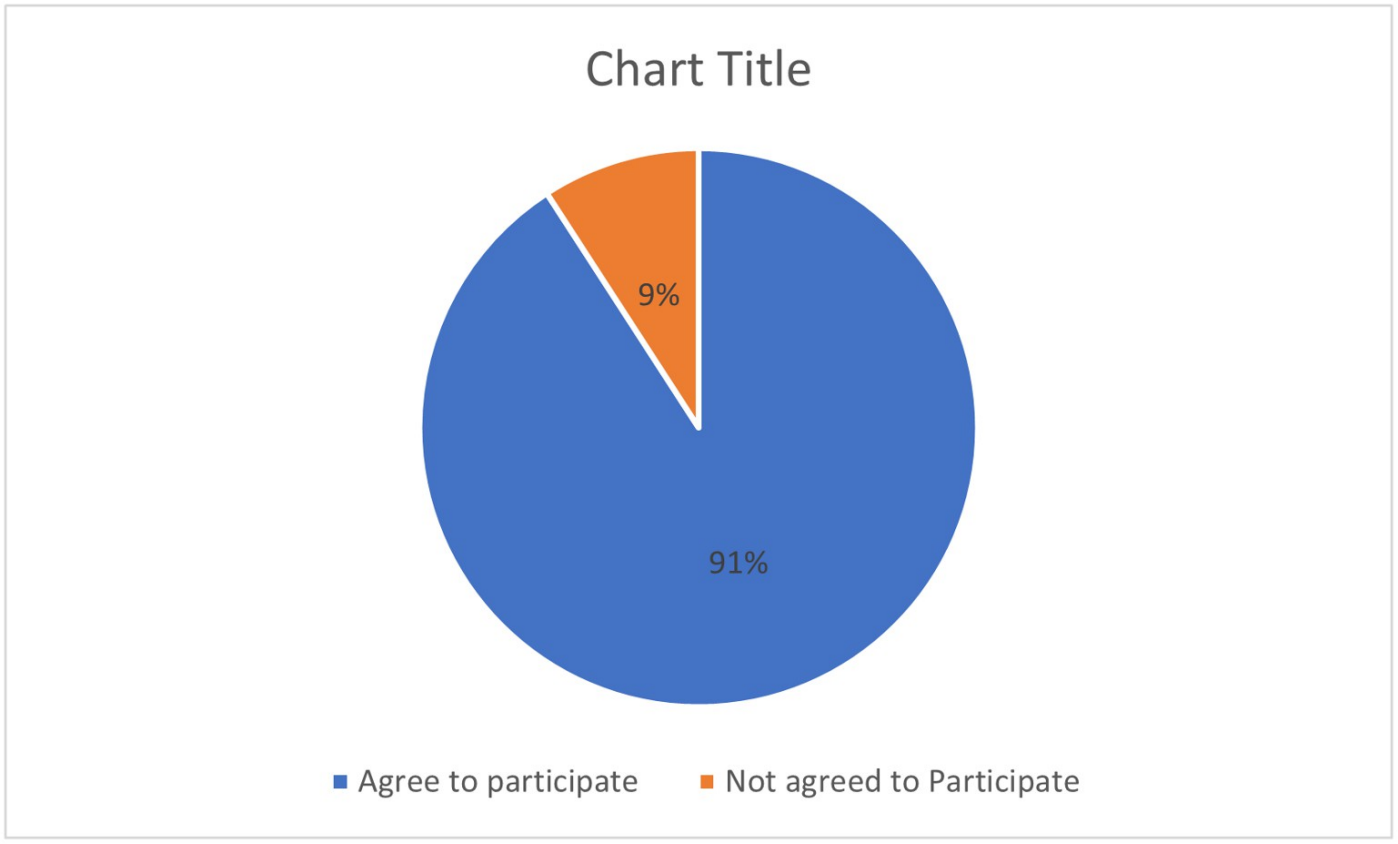
Response rate of the participants.

A total of 774 participants were participated in the study out of 852 and the response rate was 90.2% as shown in Fig 1. The mean (± standard deviation) age of the participants was 23.06 ± 4.35. The majority of the participants had single marital status (87.4%). Most of the respondents were belonging to pharmaceutical sciences (63.5%) followed by medical sciences (17.5%) and biological sciences (11.1%). Almost three-quarters of the participants were studying at the undergraduate level (74.9%) and most of the participants had rural residency (76.7%). Based on provincial distribution, the majority of the participant were belonging to Punjab (41.5%) followed by Khyber Pakhtunkhwa (28.3%) Sindh (17.1%), and Balochistan (13.1%) The detail of demographic characteristics can be seen in Table 1.

**Table 1:** Demographic characteristics of the participants.

		Mean	Standard Deviation
Age		23.06	4.35
		Frequency	Percentage
Marital status	Married	85	12.6
	Single	589	87.4
Major Discipline	Social sciences	28	4.2
	Biological sciences	75	11.1
	Pharmaceutical sciences	428	63.5
	Medical sciences	118	17.5
	other	25	3.7
Level of education	Undergraduate (UG)	505	74.9
	Post-graduate (PG)	169	25.1
Residence	Urban	157	23.3
	Rural	517	76.7
Residential Province	Khyber Pakhtunkhwa	191	28.3
	Punjab	280	41.5
	Sindh	115	17.1
	Balochistan	88	13.1

Regarding the breast cancer risk factors, most of the participants were unaware of the risk factors associated with breast cancer. only a small portion of the respondents was known to risk factors of breast cancer like the history of breast cancer in their first-degree relatives (29.8%) and benign breast disease (28%), use of oral contraceptives more than 5 years (14.1%), and after menopause use of hormone therapies (19.5%). More than one-quarter of the participants considered radiation as a risk factor for breast cancer (27.9%). Furthermore, risk factors such as a history of breast cancer (p = 0.005), benign breast illnesses (p = 0.008), hormone therapy after menopause (p = 0.009), and radiation at a younger age (p = 0.038) were found to differ significantly across educational levels. The undergraduate females were more knowledgeable about risk factors of breast cancer including the history of breast cancer in their relatives (UG: 76.2% vs PG: 23.8%) and benign diseases (UG: 79.7% vs PG: 20.3%), use hormone therapy (UG: 72.2% vs PG: 27.8%), and high exposure to radiations (UG: 76.9% vs PG: 23.1%) as shown in Table 2.

**Table 2:** Participant’s knowledge of breast cancer’s risk factors regarding the history of breast cancer and benign disease, exposure to radiations, and use of medication.

Following are the risk factors of breast cancer?		Overall	Level of education		p-value*
			Undergraduate	Post-graduate	
		Frequency (%)	Frequency (%)	Frequency (%)	
History of breast cancer in the first-degree relative	Yes	231 (29.8)	176 (76.2)	55 (23.8)	0.005
	No	446(57.6)	347 (77.8)	99 (22.2)	
	No Comment	97 (12.5)	58 (59.8)	39 (40.2)	
Use oral contraceptive pills more than 5 years	Yes	109 (14.1)	79 (72.5)	30 (27.5)	0.154
	No	507 (65.5)	394 (77.7)	113 (22.3)	
	No Comment	158 (20.4)	108 (68.4)	50 (31.6)	
Hormone therapy after menopause	Yes	151 (19.5)	109 (72.2)	42 (27.8)	0.009
	No	442 (57.1)	351 (79.4)	91 (20.6)	
	No Comment	181 (23.4)	121 (66.9)	60 (33.1)	
History of benign breast disease	Yes	217 (28.0)	173 (79.7)	44 (20.3)	0.008
	No	486 (62.8)	367 (75.5)	119 (24.5)	
	No Comment	71 (9.2)	41 (57.7)	30 (42.3)	
High radiation to the chest or breast in childhood or adolescence (radiation therapy)	Yes	216 (27.9)	166 (76.9)	50 (23.1)	0.038
	No	503 (65.0)	383 (76.1)	120 (23.9)	
	No Comment	55 (7.1)	32 (58.2)	23 (41.8)	

*The P-value represents the difference in the level of knowledge about risk factors of breast cancer between undergraduate and postgraduate students.

A small number of the participants considered early menstruation (19.1%), late menopause (16.5%), and giving birth for the first time after age 30 (14%) as risk factors for breast cancer. The female that enrolled in undergraduate programs had more knowledge about the early menstruation at age 12 as a risk factor for breast cancer (UG: 69.6% vs PG: 30.4%, p = 0.021). The detail can be seen in Table 3.

**Table 3:** Participant’s knowledge of breast cancer’s risk factors related to gynecological and obstetrics.

Following are the risk factors of breast cancer?		Overall	Level of education		p-value*
			Undergraduate	Postgraduate	
		Frequency (%)	Frequency (%)	Frequency (%)	
Started menstruating before age 12	Yes	148 (19.1)	103 (69.6)	45 (30.4)	0.021
	No	559 (72.2)	436 (78.0)	123 (22.0)	
	No Comment	67 (8.7)	42 (62.7)	25 (37.3)	
Late menopause (after age 55)	Yes	128 (16.5)	97 (75.8)	31 (24.2)	0.466
	No	331 (42.8)	255 (77.0)	76 (23.0)	
	No Comment	315 (40.7)	229 (72.7)	86 (27.3)	
Giving birth for the first time after age 30	Yes	108 (14.0)	83 (76.9)	25 (23.1)	0.506
	No	436 (56.3)	325 (74.5)	111 (25.5)	
	No Comment	230 (29.7)	173 (75.2)	57 (24.8)	
Not having a childbirth experience	Yes	361 (46.6)	270 (74.8)	91 (25.2)	0.511
	No	296 (38.2)	227 (76.7)	69 (23.3)	
	No Comment	117 (15.1)	84 (71.8)	33 (28.2)	

*The P-value represents the difference in the level of knowledge about risk factors of breast cancer between undergraduate and postgraduate students.

A poor knowledge about breast cancer’s risk factors was reported by the participants. As a small number of participants considered overweight and obesity (34.9%), lack of breastfeeding (28.3%), and low consumption of vegetables and fruits (43.5%) as the risks to breast cancer. Stress (UG: 74.4% vs PG: 25.6%, p = 0.020), high intake of red meat (UG: 71% vs PG: 29%, p = 0.035) and fatty food (UG: 75.6% vs PG: 24.4%, p = 0.008) were highly considered as risk factors for breast cancer by female undergraduate students. Participants’ Knowledge of breast cancer’s risk factors related to physical activities and lifestyle can be seen in Table 4.

**Table 4:** Participant’s knowledge of breast cancer’s risk factors related to physical activities, and lifestyle.

Following are the risk factors of breast cancer?		Overall	Level of education		p-value*
			Undergraduate	Postgraduate	
		Frequency (%)	Frequency (%)	Frequency (%)	
Low physical activity	Yes	392 (50.6)	301 (76.8)	91 (23.2)	0.199
	No	327 (42.2)	246 (75.2)	81 (24.8)	
	No Comment	55 (7.1)	34 (61.8)	21 (38.2)	
Overweight and obesity	Yes	270 (34.9)	200 (74.1)	70 (25.9)	0.602
	No	455 (58.8)	347 (76.3)	108 (23.7)	
	No Comment	49 (6.3)	34 (69.4)	15 (30.6)	
Age over 40 years	Yes	170 (22.0)	122 (71.8)	48 (28.2)	0.211
	No	540 (69.8)	417 (77.2)	123 (22.8)	
	No Comment	64 (8.3)	42 (65.6)	22 (34.4)	
Lack of breastfeeding	Yes	219 (28.3)	157 (71.7)	62 (28.3)	0.556
	No	306 (39.5)	239 (78.1)	67 (21.9)	
	No Comment	249 (32.2)	185 (74.3)	64 (25.7)	
Smoking or alcohol consumption in the past or present	Yes	163 (21.1)	113 (69.3)	50 (30.7)	0.180
	No	558 (72.1)	432 (77.4)	126 (22.6)	
	No Comment	53 (6.8)	36 (67.9)	17 (32.1)	
Stress	Yes	507 (65.5)	377 (74.4)	130 (25.6)	0.020
	No	216 (27.9)	169 (78.2)	47 (21.8)	
	No Comment	51 (6.6)	35 (68.6)	16 (31.4)	
High consumption of red meat	Yes	162 (20.9)	115 (71.0)	47 (29.0)	0.035
	No	529 (68.3)	411 (77.7)	118 (22.3)	
	No Comment	83 (10.7)	55 (66.3)	28 (33.7)	
Low consumption of vegetables and fruits	Yes	337 (43.5)	245 (72.7)	92 (27.3)	0.384
	No	391 (50.5)	305 (78.0)	86 (22.0)	
	No Comment	46 (5.9)	31 (67.4)	15 (32.6)	
High consumption of fatty foods	Yes	353 (45.6)	267 (75.6)	86 (24.4)	0.008
	No	367 (47.4)	284 (77.4)	83 (22.6)	
	No Comment	54 (7.0)	30 (55.6)	24 (44.4)	

*The P-value represents the difference in the level of knowledge about risk factors of breast cancer between undergraduate and postgraduate students.

Most of the participants were unknown to the signs and symptoms of breast cancer. The sign and symptoms like painless and palpable breast lump, painless mass under the armpit, and bleeding or discharge from the nipple were reported by 38.6%, 35.5%, and 28.4% of the participant, respectively. In addition, inward pulled nipple, wound around the nipple, and abrupt changes in the breast size were considered as the sign and symptoms by 25.2%, 25.7%, and 31.7% of the participants, respectively. There was a significant difference between undergraduate and postgraduate female participants regarding painless and palpable breast lump (UG: 75.8% vs PG: 24.2%, p = 0.011), painless mass under armpit (UG: 74.3% vs PG: 24.2%, p <0.001), and wounds around the nipple (UG: 74.7% vs PG: 25.3%, p = 0.019). The detail can be seen in Table 5.

**Table 5:** Participants knowledge of breast cancer signs and symptoms.

Following are the signs and symptoms of breast cancer?		Overall	Level of education		p-value*
			Undergraduate	Postgraduate	
		Frequency (%)	Frequency (%)	Frequency (%)	
Painless and palpable breast lump	Yes	299 (38.6)	188 (75.8)	60 (24.2)	0.011
	No	421 (54.4)	289 (76.7)	88 (23.3)	
	No Comment	54 (7.0)	28 (57.1)	21 (42.9)	
Painless mass under armpit	Yes	275 (35.5)	165 (74.3)	57 (25.7)	<0.001
	No	455 (58.8)	317 (77.9)	90 (22.1)	
	No Comment	44 (5.7)	23 (51.1)	22 (48.9)	
Bleeding or discharge from the nipple	Yes	220 (28.4)	129 (71.7)	51 (28.3)	0.131
	No	507 (65.5)	347 (77.1)	103 (22.9)	
	No Comment	47 (6.1)	29 (65.9)	15 (34.1)	
Pulling of the nipple inward	Yes	195 (25.2)	129 (75.0)	43 (25.0)	0.081
	No	492 (63.6)	329 (76.7)	100 (23.3)	
	No Comment	87 (11.2)	47 (64.4)	26 (35.6)	
Wound around the nipple	Yes	199 (25.7)	124 (74.7)	42 (25.3)	0.019
	No	513 (66.3)	349 (76.9)	105 (23.1)	
	No Comment	62 (8.0)	32 (59.3)	22 (40.7)	
Redness of the breast skin	Yes	231 (29.8)	146 (72.3)	56 (27.7)	0.229
	No	496 (64.1)	330 (76.9)	99 (23.1)	
	No Comment	47 (6.1)	29 (67.4)	14 (32.6)	
Abrupt changes in the size of the breast	Yes	246 (31.8)	147 (73.1)	54 (26.9)	0.354
	No	466(60.2)	318 (76.6)	97 (23.4)	
	No Comment	62 (8.0)	40 (69.0)	18 (31.0)	
Abrupt changes in the shape of the breast	Yes	245 (31.7)	157 (75.8)	50 (24.2)	0.317
	No	483 (62.4)	315 (75.5)	102 (24.5)	
	No Comment	46 (5.9)	33 (66.0)	17 (34.0)	

*The P-value represents the difference in the level of knowledge about signs and symptoms of breast cancer between undergraduate and postgraduate students.

Regarding the best time to start the breast exam by the doctor or midwife, only 20.9% of the participants identified the correct year for starting breast cancer examination. Most of the participants correctly favored that mammography should be initiated after 40 years (44.4%). Majority of the participant was of the opinion that breast self-examination should be started after 20 years (80%) and should perform on monthly basis (71.5%). The undergraduate female participants were more in favor to start self-breast examination (UG: 77.7% vs PG: 22.3%, p = 0.001) and mammography (UG: 80.1% vs PG: 19.9%, p = 0.021) should instructed after 20 years as shown in Table 6.

**Table 6:** Participant’s knowledge of best time to do breast examination for breast cancer.

		Overall	Level of education		p-value*
			Undergraduate	Postgraduate	
		Frequency (%)	Frequency (%)	Frequency (%)	
When is the best time to start the breast exam by the doctor or midwife?	After 20 years	375 (55.6)	283 (75.5)	92 (24.5)	0.881
	After 25 years	158 (23.4)	116 (73.4)	42 (26.6)	
	After 30 years	141 (20.9)	106 (75.2)	35 (24.8)	
When is the best time to start mammography?	After 20 years	271 (40.2)	217 (80.1)	54 (19.9)	0.021
	After 30 years	104 (15.4)	70 (67.3)	34 (32.7)	
	After 40 years	299 (44.4)	218 72.9)	81 (27.1)	
When is the best time to start self-breast exam?	After 20 years	539 (80.0)	419 (77.7)	120 (22.3)	<0.01
	After 30 years	17 (2.5)	8 (47.1)	9 (52.9)	
	After 40 years	118 (17.5)	78 (66.1)	40 (33.9)	
When is the best time to do a self-breast exam in the menstrual cycle?	One week after the onset of menstruation	415 (61.6)	311 (74.9)	104 (25.1)	0.992
	One month after the onset of menstruation	259 (38.4)	194 (74.9)	65 (25.1)	
How often should a breast cancer self-examination perform?	Monthly	482 (71.5)	368 (76.3)	114 (23.7)	0.177
	Quarterly	192 (28.5)	137 (71.4)	55 (28.6)	

*The P-value represents the difference in the level of knowledge about the best time to do breast examination for breast cancer between undergraduate and postgraduate students.

## Discussion

The current study was conducted to assess the knowledge and understandings of university female students about breast cancer. The participants reported overall poor knowledge regarding breast cancer risk factors, and signs and symptoms. Additionally, the knowledge was also poor regarding the best time for examination of breast cancer.

A good knowledge of risk factors can help in the prevention of breast cancer in the female population [26]. The history of breast cancer, in first-degree relatives, is the major risk factor for breast cancer [26]. In the current study, only 29.4% of the participants considered this as a risk factor for developing breast cancer. But in contrast, a good knowledge was reported from the female university students of Nigeria (46.4%) [27] and Uganda (48.5%) [28]. Moreover, studies published from Turkey (54.8%) [29] and China (63.6%) [30] reported a good knowledge regarding the development of breast cancer in females having a family history of this cancer. It has been reported that prolonged use of oral contraceptives (more than 5 years) increases the risk of developing breast cancer [26]. In Pakistan, majority of the population including female students don’t use contraceptive techniques and these methods are not discussed in-home and at universities. The current study results showed that only 14% of the participants marked the prolonged use of oral contraceptives as a risk factor for breast cancer. But in contrast, a study reported from Nepal showed moderate knowledge regarding the prior stated risk factor (35.7%) [31]. Moreover, only 19.1% of the participant favored early menstruation as a risk factor for breast cancer in the current study. This result was consistent with a reported study reported from Nepal, where 19.2% of the female students considered early menstruation as a risk factor for breast cancer [31]. It has been well established that breast cancer mostly develops in the older age population [3]. This risk factor was only recognized by 22% of the participants in the current study. This number was consistent with the previously reported study of Uganda university female students (25%) [28].

The early detection of breast cancer in females can only be possible when they are familiar with their signs and symptoms. In the current study, participants were little known to the signs and symptoms of breast cancer that include painless and palpable breast lump (38.6%), and painless mass under the armpit (35.5%). A comparatively higher knowledge about these signs and symptoms was reported from Ethiopia (53.7%, 57% respectively) [32] and in a similar study, painless breast lamp was correctly identified as breast cancer symptom by western Nepali higher secondary students [31], that was relatively higher as compared to our findings. Moreover, the participant in the current study was little known to the morphological changes in the breast like abrupt changes in size and shaped can be a sign and symptom of breast cancer while the awareness about this sign and symptoms was high in Sharjah (74.7%) and Ethiopian (74.3%) university female students [32, 33]. In the current study, the participant had poor knowledge regarding the signs and symptoms like wound around the nipple (25.7%) and bleeding or discharge from the nipple (28.4%). These findings were consistent with a reported study from China (37.2%, 29.4% respectively) [34].

The knowledge of screening tests for breast cancer has a vital importance in the early detection of cancer. The United States Preventive Services Task Force has recommended that women with an age range of 20–30 years should perform clinical breast examination by a healthcare provider every three years while women having age greater than 40 years should perform every year. The current study result was comparable with the recommendation as most of the participants suggested that clinical breast examination by a healthcare provider should be started after the age of 20 years (55.6%). While this frequency was low as compared to the study reported from nurses in the United Arab Emirates (96.1%). The difference in knowledge may be due to the study participants, as the study respondents were nurses which may have greater clinical experience.

The social norms of the Pakistani population have a significant effect on the participant’s knowledge. For instance, it has been reported that Pakistan women feel shyness, personal modesty, and embarrassment during the examination of breasts, especially in rural regions. The open discussion of issues, related to the breast, between women, mothers, daughters, and spouses or the extended family system is considered as a social taboo. This means that breast cancer is a socially unacceptable disease. Moreover, socioeconomic and cultural factors, such as age, employment status, lack of information, fear of surgery, trust in traditional therapies, and spiritual healing, lead the women in Pakistan to seek health facilities only at the end stages of breast cancer. Therefore, these factors are considered as a major barrier to breast cancer screening. That’s why they are diagnosed at the last stage when there is the only option of surgery [35–38]. Similarly in Iran embarrassment, fear of breast cancer diagnosis, and belief in fate were the major reported barriers by females to breast cancer screening [39]. In addition, the study conducted in Canada reported the uncomfortable feeling of women while discussing breast cancer screening as a barrier to the screening of breast cancer. The barriers to breast cancer screening, which include feeling uncomfortable while discussing screening. Pakistan is a middle-income country and 24.1% of the population lives below the national poverty line [40]. In addition, there is no program offered by the government for breast cancer education and screening [41]. Therefore, the population is unable to perform self-service screening practices of breast cancer, which also increases the risk of late diagnosis.

### Study limitation

This study was subjected to various limitations. Firstly, the study was based on the questionnaire, therefore recall biases cannot be ignored. Secondly, the study population was university female students, however, the responses may be different from the illiterate population that was not included in the current study. Thirdly, as the majority of the participants are from the biological and medical backgrounds, therefore, response bias cannot be completely neglected. Lastly, the current study utilized online data collection, where responses from the population having no access to the internet were not collected.

## Conclusion and recommendations

In summary, university female students reported a poor knowledge about breast cancer’s risk factors, signs and symptoms, and breast examination. Interestingly, although most of the participants were from pharmaceutical and medical sciences, still the knowledge was poor. It has been seen that good knowledge about breast cancer’s risk factors, signs and symptoms, and self, and clinical examination practices play an important role in the early detection of this cancer. The early diagnosis increases the chance of recovery and also improves the surveillance rate of breast cancer patients. In contrast, the current study showed poor knowledge about breast cancer that increases the risk of a diagnosis of this cancer at later stages. The late diagnosis negatively impacts the recovery and surveillance of breast cancer patients and also increases the burden on the healthcare system. Therefore, the government health administration and other related bodies (national and international) should play a role in awareness of the general public by arranging seminars. Moreover, it is suggested that awareness weeks in the educational sectors should be included in their policies. In addition, the breast cancer examination techniques should be included in their course content. Moreover, the government should encourage the health professional to spread the awareness among general population and the health facilities should be accessible to the population living in the countryside.

## Supporting information

S1 File (PDF)

## References

[1] CDC. What Is Breast Cancer? 2020 [cited 2021 4 April]. https://www.cdc.gov/cancer/breast/basic_info/what-is-breast-cancer.htm.

[2] ACS. What Is Breast Cancer? 2019 [cited 2021 4 April]. https://www.cancer.org/cancer/breast-cancer/about/what-is-breast-cancer.html.

[3] Sun Y-S, Zhao Z, Yang Z-N, Xu F, Lu H-J, Zhu Z-Y, et al. Risk Factors and Preventions of Breast Cancer. International journal of biological sciences. 2017;13(11):1387–97. doi: 10.7150/ijbs.21635 . 29209143 PMC5715522

[4] Brewer HR, Jones ME, Schoemaker MJ, Ashworth A, Swerdlow AJ. Family history and risk of breast cancer: an analysis accounting for family structure. Breast cancer research and treatment. 2017;165(1):193–200. Epub 2017/06/05. doi: 10.1007/s10549-017-4325-2 mc5511313. 28578505 PMC5511313

[5] Washbrook E. Risk factors and epidemiology of breast cancer. Women’s Health Medicine. 2006;3(1):8–14.

[6] Horn J, Vatten LJ. Reproductive and hormonal risk factors of breast cancer: a historical perspective. International journal of women’s health. 2017;9:265–72. Epub 2017/05/12. doi: 10.2147/IJWH.S129017 mc5414577. 28490905 PMC5414577

[7] Horn J, Åsvold BO, Opdahl S, Tretli S, Vatten LJ. Reproductive factors and the risk of breast cancer in old age: a Norwegian cohort study. Breast cancer research and treatment. 2013;139(1):237–43. Epub 2013/04/23. doi: 10.1007/s10549-013-2531-0 . 23605085

[8] Key TJ, Appleby PN, Reeves GK, Travis RC, Alberg AJ, Barricarte A, et al. Sex hormones and risk of breast cancer in premenopausal women: a collaborative reanalysis of individual participant data from seven prospective studies. The Lancet Oncology. 2013;14(10):1009–19. Epub 2013/07/31. doi: 10.1016/S1470-2045(13)70301-2 mc4056766. 23890780 PMC4056766

[9] Hamajima N, Hirose K, Tajima K, Rohan T, Calle EE, Heath CW Jr., et al. Alcohol, tobacco and breast cancer—collaborative reanalysis of individual data from 53 epidemiological studies, including 58,515 women with breast cancer and 95,067 women without the disease. British journal of cancer. 2002;87(11):1234–45. Epub 2002/11/20. doi: 10.1038/sj.bjc.6600596 mc2562507. 12439712 PMC2562507

[10] Jung S, Wang M, Anderson K, Baglietto L, Bergkvist L, Bernstein L, et al. Alcohol consumption and breast cancer risk by estrogen receptor status: in a pooled analysis of 20 studies. International journal of epidemiology. 2016;45(3):916–28. Epub 2015/09/01. doi: 10.1093/ije/dyv156 mc5005939. 26320033 PMC5005939

[11] Suleiman AK. Awareness and attitudes regarding breast cancer and breast self-examination among female Jordanian students. Journal of basic and clinical pharmacy. 2014;5(3):74–8. doi: 10.4103/0976-0105.139730 . 25278670 PMC4160723

[12] WHO. Breast cancer 2021 [cited 2021 4 April]. https://www.who.int/news-room/fact-sheets/detail/breast-cancer.

[13] Liu J, Wang J. Disability-Adjusted Life-Years (DALYs) for Breast Cancer and Risk Factors in 195 countries: Findings from Global Burden of Disease Study 2017. medRxiv. 2020:2020.04.02.20050534. doi: 10.1101/2020.04.02.20050534

[14] IARC. Breast cancer statistics in Pakistan 2021 [2021]. 4 April]. https://gco.iarc.fr/today/data/factsheets/populations/586-pakistan-fact-sheets.pdf.

[15] Zaheer S, Shah N, Maqbool SA, Soomro NM. Estimates of past and future time trends in age-specific breast cancer incidence among women in Karachi, Pakistan: 2004–2025. BMC Public Health. 2019;19(1):1001. doi: 10.1186/s12889-019-7330-z 31345204 PMC6659231

[16] Ahmed A, Zahid I, Ladiwala ZFR, Sheikh R, Memon AS. Breast self-examination awareness and practices in young women in developing countries: A survey of female students in Karachi, Pakistan. J Educ Health Promot. 2018;7:90-. doi: 10.4103/jehp.jehp_147_17 . 30079361 PMC6052780

[17] Noreen M, Murad S, Furqan M, Sultan A, Bloodsworth P. Knowledge and awareness about breast cancer and its early symptoms among medical and non-medical students of Southern Punjab, Pakistan. Asian Pacific Journal of Cancer Prevention. 2015;16(3). doi: 10.7314/apjcp.2015.16.3.979 25735392

[18] Sobani Z-u-A, Saeed Z, Baloch HN-u-A, Majeed A, Chaudry S, Sheikh A, et al. Knowledge attitude and practices among urban women of Karachi, Pakistan, regarding breast cancer. Journal of Pakistan Medical Association. 2012;62(11):1259. 23866428

[19] Rasool S, Iqbal M, Siddiqui A, Ahsan R, Mukhtar S, Naqvi S. Knowledge, Attitude, Practice towards Breast Cancer and Breast Self-examination among Female Undergraduate Students in Karachi, Pakistan. Journal of Advances in Medicine and Medical Research. 2019:1–11.

[20] Alshareef B, Yaseen W, Jawa W, Barnawe Y, Alshehry W, Alqethami H, et al. Breast Cancer Awareness among Female School Teachers in Saudi Arabia: A Population Based Survey. Asian Pacific journal of cancer prevention: APJCP. 2020;21(2):337. doi: 10.31557/APJCP.2020.21.2.337 32102508 PMC7332133

[21] Biswas S, Syiemlieh J, Nongrum R, Sharma S, Siddiqi M. Impact of Educational Level and Family income on Breast Cancer Awareness among College-Going Girls in Shillong (Meghalaya), India. Asian Pacific Journal of Cancer Prevention. 2020;21(12):3639–46. doi: 10.31557/APJCP.2020.21.12.3639 33369463 PMC8046293

[22] Liu N, Li P, Wang J, Guo P-p, Zhang X-h, Yang S, et al. Factors influencing breast cancer awareness: a cross-sectional study in China. Journal of Comparative Effectiveness Research. 2020;9(10):679–89. doi: 10.2217/cer-2020-0037 32648473

[23] Eysenbach G. Improving the quality of Web surveys: the Checklist for Reporting Results of Internet E-Surveys (CHERRIES). Journal of medical Internet research. 2004;6(3):e34. doi: 10.2196/jmir.6.3.e34 15471760 PMC1550605

[24] Eysenbach G. Correction: improving the Quality of web surveys: the Checklist for Reporting results of internet E-Surveys (CHERRIES). Journal of medical Internet research. 2012;14(1):e8. 10.2196/jmir.6.3.e34 PMC1550605 15471760

[25] Cuschieri S. The STROBE guidelines. Saudi J Anaesth. 2019;13(Suppl 1):S31–S4. doi: 10.4103/sja.SJA_543_18 . 30930717 PMC6398292

[26] Britt KL, Cuzick J, Phillips K-A. Key steps for effective breast cancer prevention. Nature Reviews Cancer. 2020;20(8):417–36. doi: 10.1038/s41568-020-0266-x 32528185

[27] Motilewa OO, Ekanem US, Ihesie CA. Knowledge of breast cancer and practice of self-breast examination among female undergraduates in Uyo, Akwa Ibom State, Nigeria. Int J Community Med Public Health. 2015;2(4):361–6.

[28] Godfrey K, Agatha T, Nankumbi J. Breast cancer knowledge and breast self-examination practices among female university students in Kampala, Uganda: a descriptive study. Oman Medical Journal. 2016;31(2):129. doi: 10.5001/omj.2016.25 27168924 PMC4861385

[29] Altay B, Avci IA, Rizalar S, Oz H, Meral D. Breast and cervical cancer knowledge and awareness among university students. Asian Pacific Journal of Cancer Prevention. 2015;16(5):1719–24. doi: 10.7314/apjcp.2015.16.5.1719 25773815

[30] Dinegde NG, Xuying L. Awareness of breast cancer among female care givers in tertiary cancer hospital, China. Asian Pacific journal of cancer prevention: APJCP. 2017;18(7):1977. doi: 10.22034/APJCP.2017.18.7.1977 28749635 PMC5648408

[31] Bhandari PM, Thapa K, Dhakal S, Bhochhibhoya S, Deuja R, Acharya P, et al. Breast cancer literacy among higher secondary students: results from a cross-sectional study in Western Nepal. BMC cancer. 2016;16(1):1–9. doi: 10.1186/s12885-016-2166-8 26887650 PMC4758038

[32] Gebresillassie BM, Gebreyohannes EA, Belachew SA, Emiru YK. Evaluation of Knowledge, Perception, and Risk Awareness About Breast Cancer and Its Treatment Outcome Among University of Gondar Students, Northwest Ethiopia. Frontiers in oncology. 2018;8:501-. doi: 10.3389/fonc.2018.00501 . 30456205 PMC6230991

[33] Rahman SA, Al-Marzouki A, Otim M, Khalil Khayat NEH, Yousuf R, Rahman P. Awareness about Breast Cancer and Breast Self-Examination among Female Students at the University of Sharjah: A Cross-Sectional Study. Asian Pac J Cancer Prev. 2019;20(6):1901–8. doi: 10.31557/APJCP.2019.20.6.1901 . 31244316 PMC7021607

[34] Liu L-Y, Wang Y-J, Wang F, Yu L-X, Xiang Y-J, Zhou F, et al. Factors associated with insufficient awareness of breast cancer among women in Northern and Eastern China: a case-control study. BMJ open. 2018;8(2):e018523–e. doi: 10.1136/bmjopen-2017-018523 . 29463589 PMC5855304

[35] Banning M, Hafeez H. A Two-Center Study of Muslim Women’s Views of Breast Cancer and Breast Health Practices in Pakistan and the UK. Journal of Cancer Education. 2010;25(3):349–53. doi: 10.1007/s13187-010-0051-8 20146040

[36] Memon ZA, Shaikh AN, Rizwan S, Sardar MB. Reasons for patient’s delay in diagnosis of breast carcinoma in Pakistan. Asian Pacific Journal of Cancer Prevention. 2013;14(12):7409–14. doi: 10.7314/apjcp.2013.14.12.7409 24460311

[37] Banning M, Hassan M, Faisal S, Hafeez H. Cultural interrelationships and the lived experience of Pakistani breast cancer patients. European Journal of Oncology Nursing. 2010;14(4):304–9. doi: 10.1016/j.ejon.2010.05.001 20584625

[38] Khaliq IH, Mahmood HZ, Sarfraz MD, Gondal KM, Zaman S. Pathways to care for patients in Pakistan experiencing signs or symptoms of breast cancer. The Breast. 2019;46:40–7. doi: 10.1016/j.breast.2019.04.005 31075671

[39] Khazir Z, Morowatisharifabad MA, Vaezi A, Enjezab B, Yari F, et al. (2019) Perceived behavioral control in mammography: a qualitative study of Iranian women’s experiences. International Journal of Cancer Management 12.

[40] bank W. Literacy rate, adult total (% of people ages 15 and above)—Pakistan 2017 [cited 2021 1 June]. https://data.worldbank.org/indicator/SE.ADT.LITR.ZS?locations=PK.

[41] Maqsood B, Zeeshan MM, Rehman F, Aslam F, Zafar A, Syed B, et al. Students’ corner breast cancer screening practices and awareness in women admitted to a Tertiary Care Hospital of Lahore, Pakistan. JPMA. 2009;59(418). 19534385

