# Supplementary material for: A national survey to assess breast cancer awareness among the female university students of Pakistan
Source: PLoS One. 2022 Jan 21;17(1):e0262030. doi: 10.1371/journal.pone.0262030 (PMC8782286; doi:10.1371/journal.pone.0262030)
Supplement: S1 File — (PDF) [file pone.0262030.s001.pdf]

# A national survey to assess breast cancer awareness among the female university students of Pakistan

## Study tool

| **Demographic characteristics of the participants** | | |
|---|---|---|
| What is your age (years) | | |
| What is your marital status | | Single<br>married |
| What is your major discipline? | | Social sciences<br>Pharmaceutical sciences<br>Biological sciences<br>Medical sciences<br>Other |
| What is your highest level of education | | Undergraduate<br>Postgraduate |
| What is your residence | | Urban<br>Rural |
| What is your residential province? | | Khyber Pakhtunkhwa<br>Punjab<br>Sindh<br>Balochistan |
| **Breast cancer knowledge** | | |
| **Risk factors for Breast cancer** | | |
| **History of breast cancer and benign disease, exposure to radiations, and use of medication** | | **Responses** |
| D1 | History of breast cancer in the first-degree relative | Yes No No comment |
| | Use oral contraceptive pills more than 5 years | Yes No No comment |
| | Hormone therapy after menopause | Yes No No comment |
| | History of benign breast disease | Yes No No comment |
| | High radiation to the chest or breast in childhood or adolescence (radiation therapy) | Yes No No comment |

| **Breast cancer's risk factors related to gynecological and obstetrics.** | | **Responses** |
|---|---|---|
| D2 | Started menstruating before age 12 | Yes No No comment |
| | Late menopause (after age 55) | Yes No No comment |
| | Giving birth for the first time after age 30 | Yes No No comment |
| | Not having a childbirth experience | Yes No No comment |
| **Breast cancer's risk factors related to physical activities, and lifestyle** | | **Responses** |
| D3 | Low physical activity | Yes No No comment |
| | Overweight and obesity | Yes No No comment |
| | Age over 40 years | Yes No No comment |
| | Lack of breastfeeding | Yes No No comment |
| | Smoking or alcohol consumption in the past or present | Yes No No comment |
| | Stress | Yes No No comment |
| | High consumption of red meat | Yes No No comment |
| | Low consumption of vegetables and fruits | Yes No No comment |
| | High consumption of fatty foods | Yes No No comment |
| **Breast cancer signs and symptoms** | | **Responses** |
| Following are the signs and symptoms of breast cancer? | | Yes No No comment |
| Painless and palpable breast lump | | Yes No No comment |
| Painless mass under armpit | | Yes No No comment |
| Bleeding or discharge from the nipple | | Yes No No comment |
| Pulling of the nipple inward | | Yes No No comment |
| Wound around the nipple | | Yes No No comment |
| Redness of the breast skin | | Yes No No comment |
| Abrupt changes in the size of the breast | | Yes No No comment |
| Abrupt changes in the shape of the breast | | Yes No No comment |
| **Best time to do breast examination for breast cancer** | | **Responses** |
| When is the best time to start the breast exam by the doctor or midwife? | | After 20 years<br>After 25 years |

| | |
|---|---|
| | After 30 years |
| When is the best time to start mammography? | After 20 years<br>After 30 years<br>After 40 years |
| When is the best time to start self-breast exam? | After 20 years<br>After 30 years<br>After 40 years |
| When is the best time to do a self-breast exam in the menstrual cycle? | One week after the onset of menstruation<br>One month after the onset of menstruation |
| How often should a breast cancer self-examination perform? | Monthly<br>Quarterly |
